# Ligand placement based on prior structures: the guided ligand-replacement method

**DOI:** 10.1107/S1399004713030071

**Published:** 2013-12-25

**Authors:** Herbert E. Klei, Nigel W. Moriarty, Nathaniel Echols, Thomas C. Terwilliger, Eric T. Baldwin, Matt Pokross, Shana Posy, Paul D. Adams

**Affiliations:** a Physical Biosciences Division, Lawrence Berkeley National Laboratory, Berkeley, CA 94720, USA; b Research and Development, Bristol-Myers Squibb, Princeton, NJ 08543-4000, USA; c Los Alamos National Laboratory, Los Alamos, NM 87545-0001, USA; d Natural Discovery LLC, Princeton, NJ 08542-0096, USA; e Department of Bioengineering, University of California at Berkeley, Berkeley, CA 94720-1762, USA

**Keywords:** ligand placement, guided ligand-replacement method, *GLR*

## Abstract

The process of iterative structure-based drug design involves the X-ray crystal structure determination of upwards of 100 ligands with the same general scaffold (*i.e.* chemotype) complexed with very similar, if not identical, protein targets. In conjunction with insights from computational models and assays, this collection of crystal structures is analyzed to improve potency, to achieve better selectivity and to reduce liabilities such as absorption, distribution, metabolism, excretion and toxicology. Current methods for modeling ligands into electron-density maps typically do not utilize information on how similar ligands bound in related structures. Even if the electron density is of sufficient quality and resolution to allow *de novo* placement, the process can take considerable time as the size, complexity and torsional degrees of freedom of the ligands increase. A new module, *Guided Ligand Replacement* (*GLR*), was developed in *Phenix* to increase the ease and success rate of ligand placement when prior protein–ligand complexes are available. At the heart of *GLR* is an algorithm based on graph theory that associates atoms in the target ligand with analogous atoms in the reference ligand. Based on this correspondence, a set of coordinates is generated for the target ligand. *GLR* is especially useful in two situations: (i) modeling a series of large, flexible, complicated or macrocyclic ligands in successive structures and (ii) modeling ligands as part of a refinement pipeline that can automatically select a reference structure. Even in those cases for which no reference structure is available, if there are multiple copies of the bound ligand per asymmetric unit *GLR* offers an efficient way to complete the model after the first ligand has been placed. In all of these applications, *GLR* leverages prior knowledge from earlier structures to facilitate ligand placement in the current structure.

## Abstract 2

A new module, *Guided Ligand Replacement (GLR)*, has been developed in *Phenix* to increase the ease and success rate of ligand placement when prior protein-ligand complexes are available.

## 1. Introduction   

### 1.1. Issues related to ligand fitting   

Ligand fitting has traditionally been one of the more problematic steps in the refinement of protein–ligand complexes. There are two reasons for this difficulty: (i) the generation of accurate parameters for refining the ligand geometry requires chemical knowledge and takes time, and (ii) the placement of the ligand in electron density can be labor-intensive and ambiguous. The first issue, ligand parameterization, has largely been addressed by current tools such as *eLBOW*/*REEL* (Moriarty *et al.*, 2009 ▶), *PRODRG* (Schüttelkopf & van Aalten, 2004 ▶), *grade* (Smart *et al.*, 2011 ▶) and *JLigand* (Lebedev *et al.*, 2012 ▶). These programs represent significant improvements over previous approaches such as *XPLO*2*D* (Kleywegt, 1995 ▶), in which all bonds of the same type (*e.g.* a single carbon–carbon bond) were parameterized identically. These newer tools often use the Cambridge Structural Database/*Mogul* (Bruno *et al.*, 2004 ▶) and quantum-mechanical calculations to provide robust parameterization with only the chemical structure as input. Given these higher quality parameter sets, it is now reasonable to expect the accuracy of the bond-length, bond-angle and torsion basis set to exceed the fidelity of the diffraction experiment. The second issue, ligand placement, is not always straightforward, especially when only partial electron density is present. Even when the density is complete, placement can be tedious for ligands with many torsional degrees of freedom and systems with several copies in the asymmetric unit.

### 1.2. Manual ligand fitting   

Graphics packages such as *QUANTA* (Oldfield, 2001*a* ▶) and *Coot* (Emsley *et al.*, 2010 ▶) have tools to fit ligands manually. Once coordinate and parameter files have been imported, graphics programs enable the crystallographer to interactively place and adjust ligands with various degrees of ease and sophistication. As a widely used graphics program, *Coot* offers many GUI-based commands to perform all the requisite steps: rotating and translating ligands as rigid bodies to roughly overlay the electron density, adjusting torsion angles to adapt the ligand conformation to the density, superimposing structures, cutting and pasting structure fragments to complete the asymmetric unit and finally real-space refining the ligand once fundamentally placed manually through the use of these tools. As a step towards automation, from within *QUANTA*, *X-­LIGAND* (Oldfield, 2001*b* ▶) could be used to automatically fit ligands with some success, albeit slowly for flexible ligands and still through an interactive session.

### 1.3. Automated ligand fitting   

The growth of structural genomics and fragment-based efforts prompted the development of refinement pipelines by both the NIH-funded Structural Genomics Centers (Chance *et al.*, 2002 ▶) and biotechnology companies (Badger, Sauder *et al.*, 2005 ▶). As one component of these pipelines, automated ligand fitting, in which the above manual steps are performed programmatically, was approached in a number of ways. A robust pipeline was created by Structural GenomiX (San Diego, California, USA; now part of Eli Lilly & Co.). The SGX pipeline used *OMEGA* (OpenEye Scientific Software, Santa Fe, New Mexico, USA; Hawkins *et al.*, 2010 ▶) to create sets of distinct conformers and then used two approaches to fit ligands (Badger, Hanson *et al.*, 2005 ▶): (i) an evolutionary algorithm to explore torsional space and score against electron-density values at the atomic positions and (ii) comparison of the convolution of the experimental ligand difference density and calculated electron density at the same resolution for each conformer. *AFITT* (OpenEye Scientific Software, Santa Fe, New Mexico, USA; Wlodek *et al.*, 2006 ▶) extended this approach to sample ligand conformations more quickly and to place the best fits. Astex Pharmaceuticals (Cambridge, England/Dublin, California, USA) also developed an effective structure pipeline geared toward fragments (Blundell *et al.*, 2002 ▶; Mooij *et al.*, 2006 ▶). Within *Phenix* (Adams *et al.*, 2002 ▶, 2010 ▶), the *LigandFit* module (Terwilliger *et al.*, 2006 ▶) was developed for *de novo* ligand fitting. This method divides the ligand into fragments with limited torsional freedom and then systematically examines ways to position these fragments into electron density and still satisfy the required chemical connectivity. *LigandFit* can be run in several modes to place a ligand in unmodeled electron density: (i) fit the ligand in the largest blob, (ii) fit the ligand near the specified residue or Cartesian coordinates (*i.e.* find-near mode) and (iii) fit the ligand wherever it can be fitted (*i.e.* find-all mode). *LigandFit* can be run either standalone (*phenix.ligandfit*) or as part of the pipeline (*phenix.ligand_pipeline*) (Echols *et al.*, 2013 ▶).

### 1.4. Limitations of *de novo* placement   

Despite the full complement of tools in *Coot* and the utility of automated approaches, *de novo* methods may not always be the best approach to ligand fitting because they require unambiguous electron density. When the current complex is expected to be similar to one or more previous structures, and when low resolution or poor map quality hinder the independent placement of each copy of the ligand, it is often advantageous to fit related ligands in subsequent structures with the aid of prior knowledge. This advantage is more pronounced when the ligand is large with many torsional degrees of freedom or contains pseudo-symmetry, or when there are several copies of the ligand in the asymmetric unit. A fit based on prior knowledge, albeit necessarily biased by this information, allows the crystallographer to quickly arrive at a potential initial placement of the ligand irrespective of these limitations. Current automated methods are handicapped because they mainly rely on the density associated with the current structure in isolation without the full benefit of chemical sense, hydrogen bonds, contacts and any other prior knowledge brought to bear by the crystallographer. Here, we describe the implementation of this method, termed *Guided Ligand Replacement* (*GLR*), within *Phenix* to utilize previously solved X-ray crystal structures of protein–ligand complexes to complete the refinement of related complexes.

## 2. Methods   

### 2.1. Overview   

**Figure 1:**
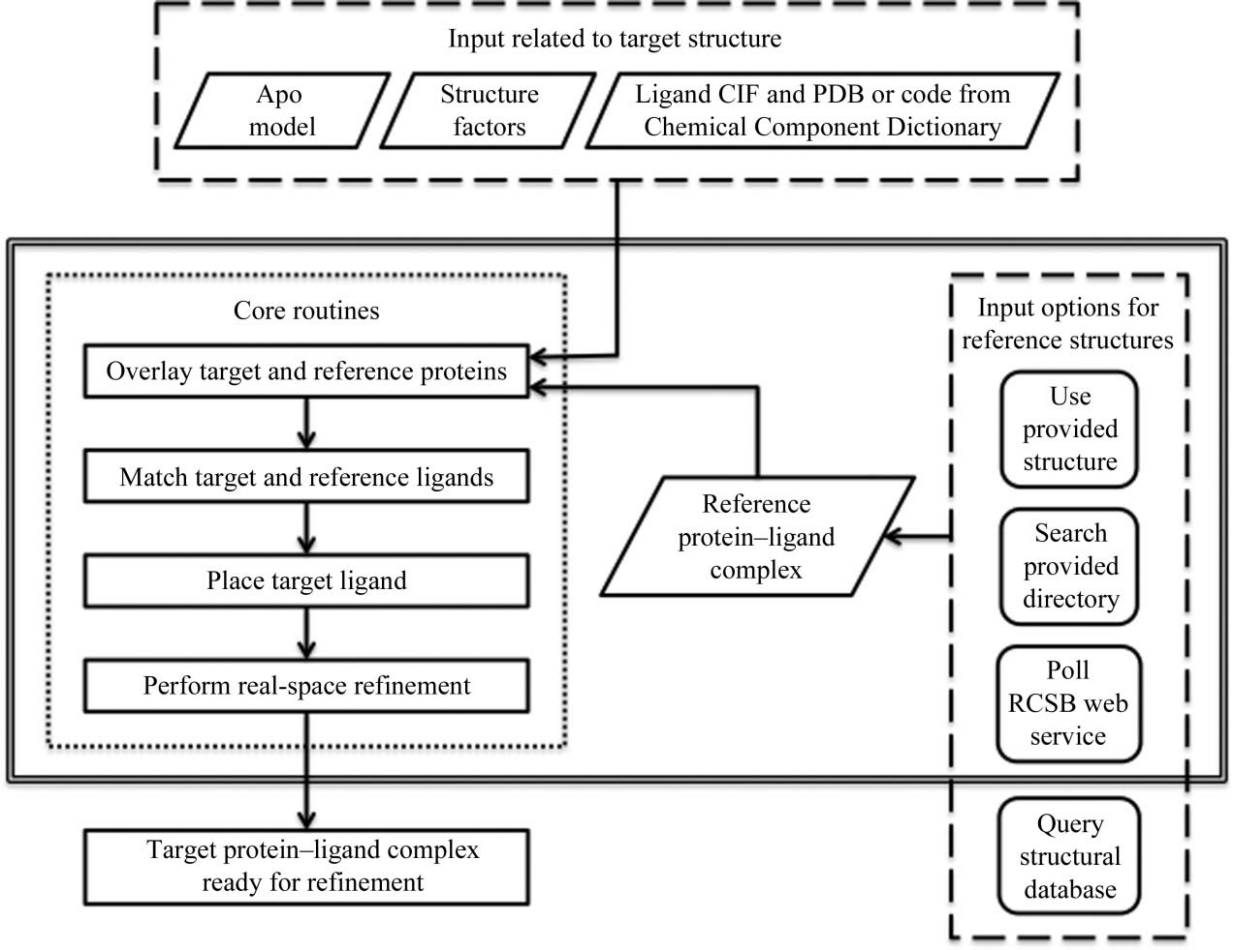
Overview of *Guided Ligand Replacement* (*GLR*) as implemented in *Phenix*. Core routines are outlined by short dashes. Inputs are outlined by long dashes. The top box lists input related to the target structure: (i) the apo model into which the ligand is to be placed (*e.g.* molecular-replacement solution), (ii) structure factors in order to generate an electron-density map for real-space refinement and (iii) ligand specification. The right box lists ways in which the structure of the reference protein–ligand complex can be obtained: (i) provide it directly, (ii) provide a directory of protein–ligand complexes to be searched, (iii) poll the Chemical Component Dictionary through the RCSB web service and (iv) a user-written query to another structural database (*e.g.* a proprietary database in the pharmaceutical industry). The functionality outlined by double lines is part of the *Phenix* distribution.

As soon as the initial difference electron density for the target ligand has been confirmed, the aim of the crystallo­grapher is to promptly model this density, complete the refinement and derive useful insights from the structure. Provided that the structure of at least one protein–ligand complex of the same or a similar protein associated with the same or a similar ligand (*i.e.* a reference structure) is available, rather than proceed with one of the *de novo* methods, *GLR* can be employed (Fig. 1 ▶). All other input (*e.g.* the initial apo model, structure factors and ligand description) is no different from what is customarily required for the structure determination of any protein–ligand complex. For cases in which the ligand to be placed (*i.e.* the target ligand) is similar to a ligand in an already refined structure (*i.e.* a reference ligand), one needs to only superimpose the proteins, establish an equivalence between atoms in the reference and the target ligands, generate a conformation of the target ligand that is consistent with this equivalence and place the target ligand throughout the asymmetric unit. The approach was implemented as follows: (i) associate each copy of the reference ligand with one protein molecule in the reference structure; (ii) superimpose the representative protein molecule from the reference structure associated with the reference ligand on analogous protein molecules in the target structure; (iii) modify the target ligand coordinates to match the selected instance of the reference ligand; (iv) apply the protein transformation matrices to the target ligand to populate the target asymmetric unit; (v) delete copies of the ligand as needed to eliminate overlapped ligands; and (vi) perform real-space refinement on the placed ligands.

### 2.2. Selection of reference structure   

**Figure 2:**
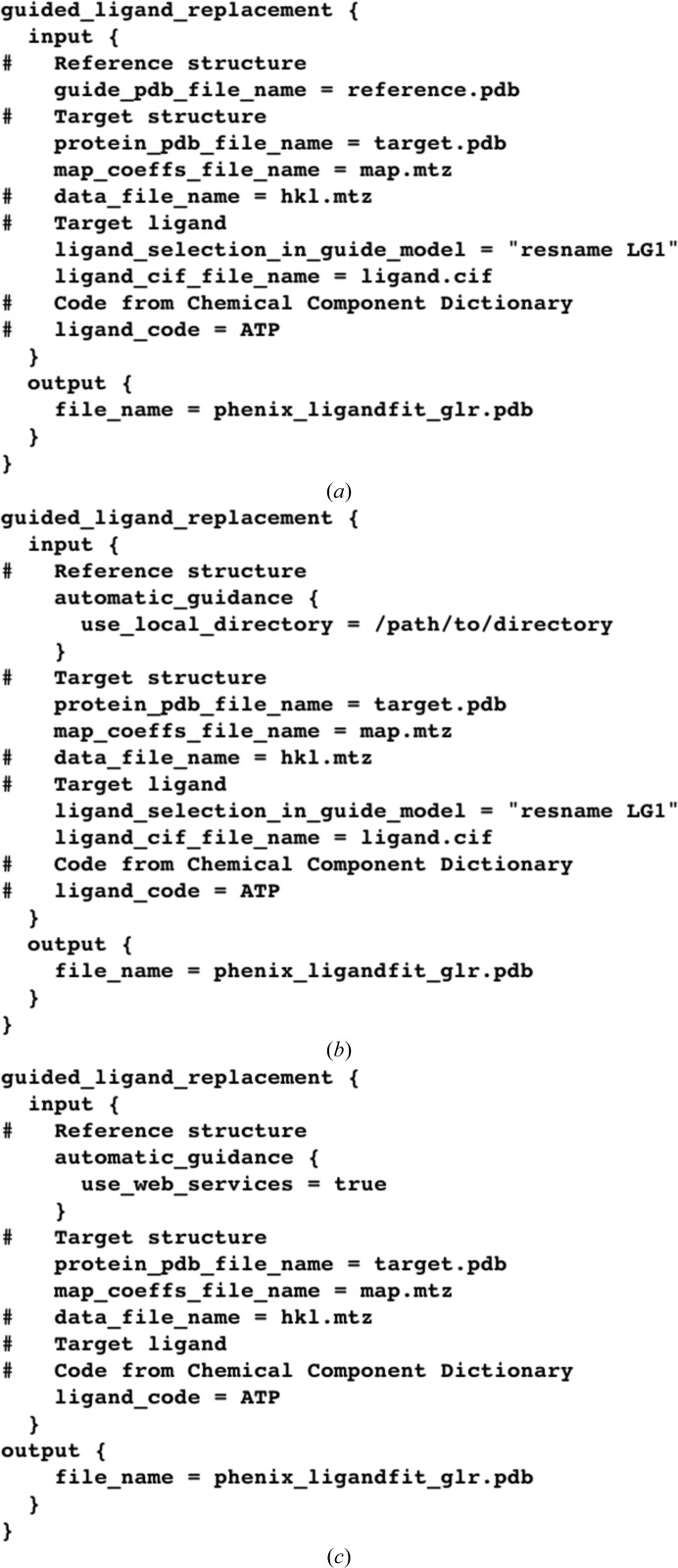
Examples of PHIL instructions for the three supported ways to arrive at the *GLR* reference structure (Fig. 1 ▶). (*a*) Provide explicitly. (*b*) Search provided directory. (*c*) Poll RCSB web service. As with other *Phenix* applications, arguments can also be provided on the command line. Online documentation for *GLR* can be found at http://www.phenix-online.org/documentation/GLR.htm.

**Figure 3:**
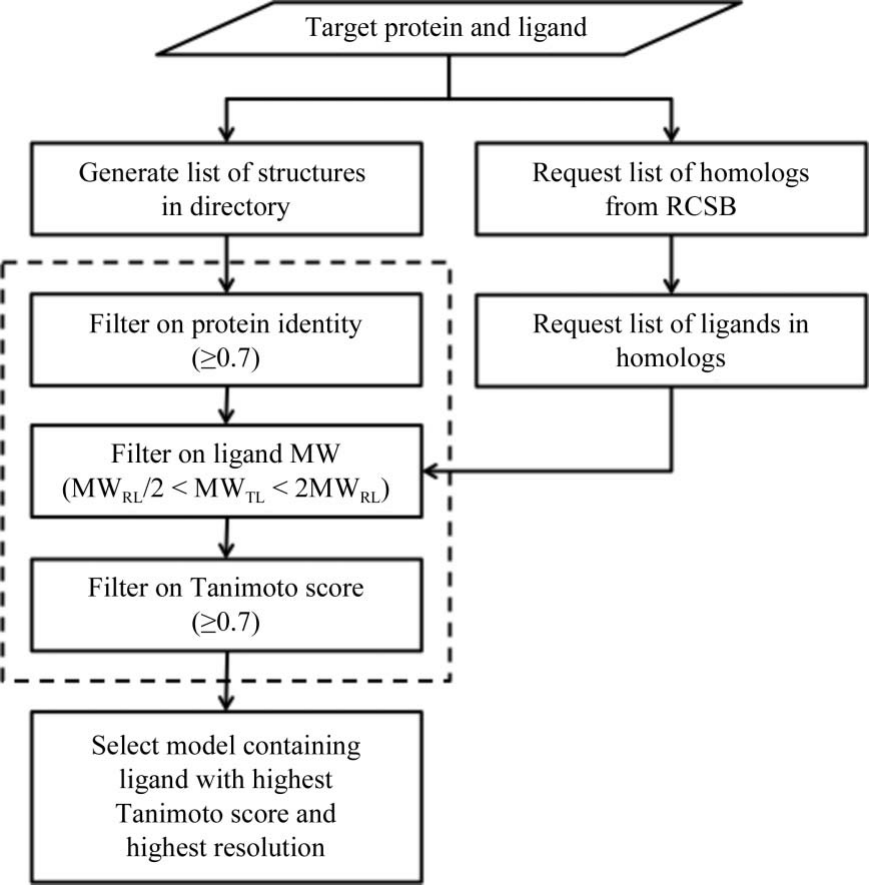
Two procedures were implemented to arrive at the reference structure when one is not explicitly specified: (i) search a provided directory and (ii) query the RCSB. While the number of structures to consider can be large, the steps outlined by the dashed box can optionally be performed in parallel. The molecular-weight filter requires each potential reference ligand (RL) to be similar in size to the input target ligand (TL).

*Phenix* supports three ways to arrive at the reference structure. Firstly, the reference model can be provided explicitly. The choice can be as straightforward as another crystal form of the same protein–ligand complex encountered earlier or facilitated with resources such as the JCSG Ligand Search Server (Kumar *et al.*, 2010 ▶; http://smb.slac.stanford.edu/jcsg/Ligand_Search). Secondly, all structures contained in a directory can be evaluated as suitable reference models (*e.g.* a directory of related kinase structures). Thirdly, the RCSB web service (Rose *et al.*, 2011 ▶) can be used to query the Chemical Component Dictionary (Henrick *et al.*, 2008 ▶) for ligands and their associated structures based on just the ligand code (*e.g.* ATP). How to obtain the reference structure is easily specified by Python-based Hierarchical Interchange Language (PHIL) instructions (Grosse-Kunstleve *et al.*, 2005 ▶) or at the command line (Fig. 2 ▶). When the local directory or web services route is specified, the reference structure must be selected from many choices. While the internal procedures used for each of the three options differ slightly (Fig. 3 ▶), the objective is the same: to identify a suitable reference structure and continue as if it had been entered explicitly. Whether or not these procedures identify the best reference structure is subject to interpretation (*i.e.* preferred tradeoffs between protein identity, ligand similarity and diffraction resolution). Multiple suitable reference structures often exist, any one of which could be used to complete the target structure. Rather than go to extraordinary lengths to identify the best reference structure, the procedures have been shown to identify viable reference structures across many test cases without undue deliberation.

### 2.3. Selection of reference ligand   

When the reference structure is specified, the reference ligand is also usually specified (*e.g.* ligand_selection_in_guide_model = ‘resname LG1 and resid 1 and chain L’, ligand_code = ‘ATP’). However, if not, the reference ligand is deduced as follows. The reference structure is checked for non-protein residues, with common residues (*e.g.* HOH, SO4) excluded. If multiple choices exist, then binary strings, or fingerprints, are calculated for the target ligand and for each potential reference ligand as per the PubChem methodology (ftp://ftp.ncbi.nlm.nih.gov/pubchem/specificitions/pubchem_fingerprints.pdf). The Tanimoto coefficient (Rogers & Tanimoto, 1960 ▶) is one way to quantify the similarity between two fingerprints [0 (no compared features match) ≤ Tanimoto coefficient ≤ 1 (all compared features match)]. The same molecule compared with itself (*i.e.* identical molecules) will have a Tanimoto coefficient of 1; however, the converse is not always true. A Tanimoto coefficient of 1 does not guarantee identity because features of the two molecules that have not been encoded in the fingerprint could differ. The reference ligand is taken as the residue (*i.e.* molecule) most similar to the target ligand based on the highest Tanimoto coefficient. Even though ligands are normally entered as HETATM records, ATOM records are examined as well to accommodate the vagaries of other software and refinement practices. Each copy of the reference ligand is then associated with one protein chain in the reference structure. This association is assigned as the protein chain with the shortest interatomic distance from any atom in the protein chain to any atom in the ligand. Finally, the first complete, or most complete, instance of the ligand based on the number of heavy (*i.e.* non-H) atoms is selected as the reference ligand.

### 2.4. Atomic correspondence between reference and target ligands   

**Figure 4:**
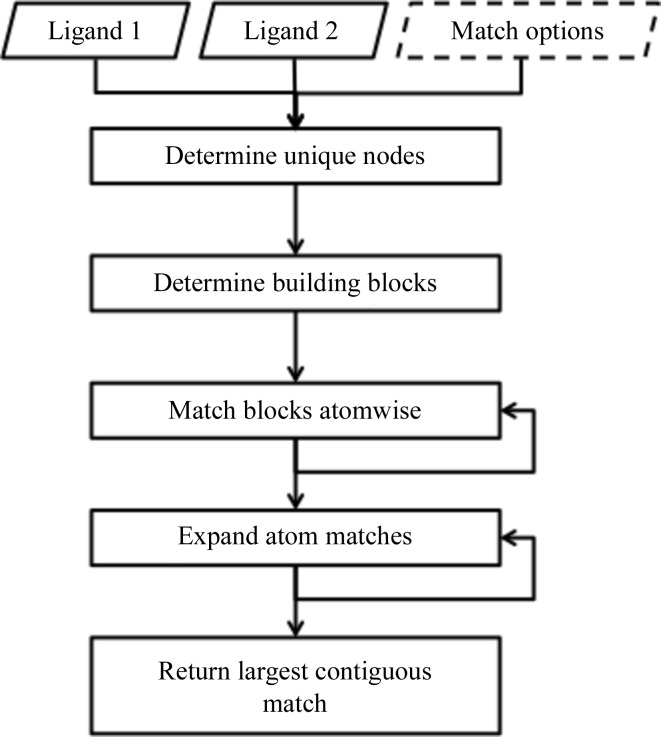
Overview of the matching algorithm used in *eLBOW* to associate analogous atoms in two molecules. The input consists of the two ligands in any accepted format (*e.g.* SDF). Unlike routines such as *phenix.superpose_ligands*, in *GLR* the match options are fixed and not exposed to the user through PHIL instructions. Both the matching of building blocks and the expansion of atom matches are iterative. These steps are repeated to generate the greatest number of atomic matches.

Once the reference ligand has been determined, an atom-based association between it and the target ligand is required. The *eLBOW* module in *Phenix* has a very flexible procedure for matching two ligands through several graph-matching techniques and *ad hoc* matching algorithms (Fig. 4 ▶). At the core is an iterative matching algorithm that uses various moieties as graph nodes. Ligands are analyzed for building blocks such as rings and key atoms from which multiple branches extend. The building blocks of two ligands are compared using a matching algorithm based on nodes and edges. Unique nodes are first determined in order to assist with the determination of larger blocks. This procedure is especially flexible because it is able to handle H atoms, or the lack thereof, on either block. The subgraph isomorphism algorithm (Cordella *et al.*, 2004 ▶) was modified to the application of almost planar graphs. In the case of multiple matches of building blocks, the Hopcroft–Karp method (Hopcroft & Karp, 1973 ▶) is used to make a selection. In the case of identical selections, one is chosen at random. Once the building blocks have been matched, connected atoms are iteratively matched. After each iteration, the set of new matches is tested for consistency with the existing set before being added. In terms of computational complexity theory, subgraph isomorphisms are NP-complete problems and, as such, these problems are only amenable to timely solution through the use of approximations (*e.g.* randomization). Because the Hopcroft–Karp method depends on atom order, randomization is inherent in the connected atom matching. Owing to this random component, the atoms in each molecule are arbitrarily reordered and the procedure is repeated three times to help to ensure the best match. Matched atoms are superposed directly onto the reference atoms. Unmatched atoms in the target ligand are tagged for later positioning using the simple force-field optimization methods in *eLBOW* when inserted into the target model.

### 2.5. Superposition of reference and target structures and target-ligand placement   

With a realistic conformation based on the reference ligand established, the target ligand can be placed in the binding site. For each chain in the target model, the reference protein chain is repositioned using the algorithms in *phenix.superpose_pdbs* (http://www.phenix-online.org/documentation/superpose_pdbs.htm). The procedure is based on the least-squares superposition of two selected parts from two PDB files; the default is the C^α^ atoms for the entire protein in each model. If the coordinate r.m.s.d. is less than 1.5 Å for matched protein residues, the rotation matrix of the protein-chain alignment is applied to the reference ligand and the associated atoms of the target ligand are superposed. As ligands are associated with protein chains in the reference model based on distance criteria, ligands that bind in sites formed by multiple protein chains (*e.g.* the HIV-1 protease homodimer) may be placed at the same site more than once. In these cases, the best-fitting ligand based on density values at the atomic centers and the density correlation of the electron clouds is retained.

### 2.6. Real-space refinement of placed target ligand   

If the target ligand was defined other than with a CIF restraints file, *GLR* calls *eLBOW* internally to compute the necessary topology and restraints. All chemical input formats accepted by *eLBOW* (*e.g.* SMILES strings; Weininger, 1988 ▶) can also be input to *GLR* to compute the target-ligand topology and restraints. Alternatively, to speed up the process, the restraints CIF file can be pre-calculated (*e.g.* computed when putative co-crystals are harvested and stored in a repository for later use). A real-space refinement is the final step in the ligand positioning and involves the surrounding protein to allow for protein deformation owing to differences between the reference and target ligands. If a map is provided, the structure is refined against it. If the map coefficient file contains more than one set of possible coefficients, the first recognized set is used. If structure factors are provided, a 2*F*_o_ − *F*_c_ map is calculated and used. Since *GLR* was designed to provide functionality related to ligand placement, the option to perform crystallographic refinement was omitted for two reasons. Firstly, it is often desirable to inspect structures after automated ligand placement, whether performed by *GLR* or otherwise, prior to crystallographic refinement. Secondly, the *GLR* module is easily integrated with refinement pipelines in which the process can continue past ligand placement to refinement without interruption.

## 3. Results and discussion   

### 3.1. Overall experience and philosophy   

Because limiting false negatives (*i.e.* failures owing to an inappropriate reference structure or to a too dissimilar reference ligand) was viewed as more important than ensuring that *GLR* always ran to completion, conservative defaults (*e.g.* a Tanimoto coefficient of 0.7 between the fingerprints of the reference and the target ligands) were used. Especially in the context of refinement pipelines, it was considered preferable to turn to *de novo* methods to fit ligands when uncertain about the applicability of *GLR*. Based on many tests, our observation was that when equivalence can be established between atoms in the reference and target ligands, and when the reference and target proteins can be reliably superimposed, *GLR* rapidly and accurately populated the entire asymmetric unit. The decision was made to not cull placed ligands based on poor real-space correlation with electron density alone. Even though this decision will result in the forced placement of ligands into what are unintentional apo structures, it was considered easier to delete erroneous ligands upon visual inspection after refinement than needing to rerun *GLR* with more tolerant correlation thresholds.

### 3.2. Summary of sample cases   

**Figure 5:**
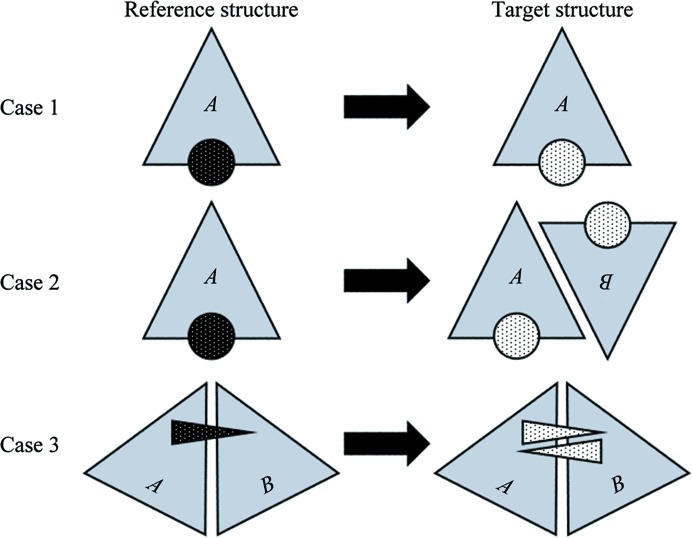
Schematic representation of three test cases, from simple to complex, in which the reference structure was used to place the ligand(s) in the target structure by *GLR*. The triangles labeled *A* and *B* represent protein molecules in the asymmetric unit. The black spheres and triangle with white dots represent ligands in the reference structures. The white spheres and triangles with black dots represent ligands in the target structures. In case 3 triangles rather than spheres were used to represent the ligands because when an asymmetric ligand is bound in an active site shared across a homomeric interface, an issue of directionality arises. The lower triangle associated with the target structure indicates the presence of an alternate ligand conformation with the reverse orientation.

Three test cases, from simple to complex, are presented to demonstrate the utility of the *GLR* method (Fig. 5 ▶). While none of these structures were originally refined with the aid of *GLR*, they illustrate practical applications of this method. In case 1, the reference and target structures are of the same crystal form with only one protein–ligand complex in the asymmetric unit. While this case is somewhat trivial because one C^α^ superposition would have provided the same protein alignment and placement of the reference ligand, *GLR* accurately morphed and placed the target ligand without manual intervention. In case 2, the structures are of different crystal forms with different numbers of protein–ligand complexes in the asymmetric units. This case raises two issues: (i) the need to apply *GLR* until all of the active sites of the target asymmetric unit are populated and (ii) the question of which copy of the reference ligand to use when multiple choices are available. In case 3, the structures belong to the same crystal form but the protein sequences are not identical and the active site is shared across a homodimer. This case demonstrates the need to eliminate overlapped ligands. While the use of the shortest intermolecular distance alone to associate a reference ligand with a protein chain in the reference structure worked well in these test cases, it is potentially problematic in unusual situations (*e.g.* a copy of the ligand bound away from the traditional site in the reference structure owing to some fortuitous feature of the crystal packing). In such cases, protein–ligand interactions could be analyzed based on the nature of the intermolecular contacts and one example of each type of interaction could be probed in the target structure. Alternatively, if the reference structure conforms to v.3.X of the PDB standard (http://www.wwpdb.org/procedure.html#toc_4), the protein chain associated with each ligand could be taken as input. Even though some of these test cases contained more than one copy of the same ligand, they all involved only one unique ligand. Successive *GLR* runs can handle structures with multiple distinct ligands.

### 3.3. Case 1: same crystal form (one molecule in asymmetric unit of reference→one molecule in asymmetric unit of target); similar ligand   

**Figure 6:**
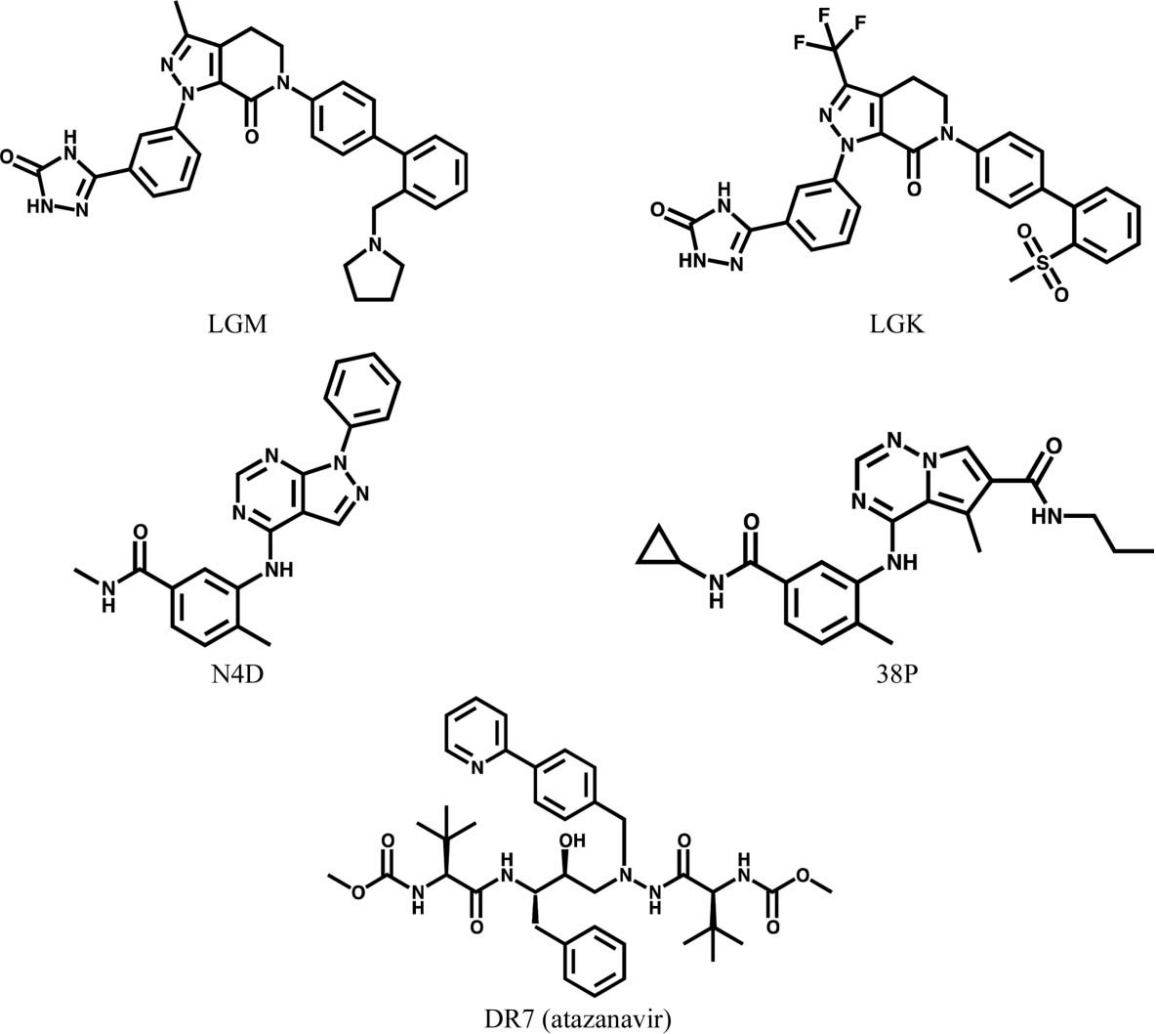
Chemical structures of the ligands LGM, LGK, N4D, 38P and DR7 (atazanavir).

**Figure 7:**
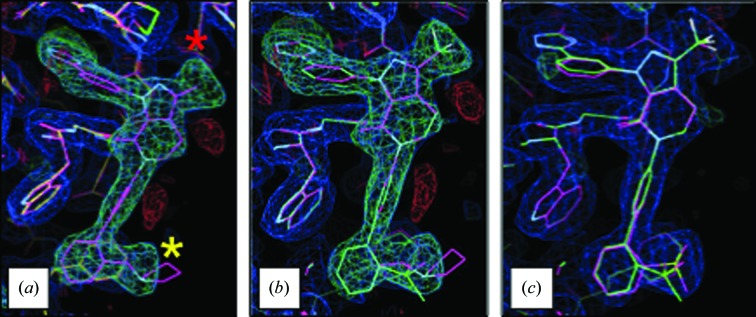
*GLR* progression for case 1 (FXa). (*a*) Superposition of the reference heavy chain (chain *A*; cyan) on the target heavy chain (chain *A*; yellow). This superposition places the reference ligand LGM (cyan) in the difference electron density for the target ligand LGK. While the fit to the electron density is reasonable, disagreement is apparent where the chemical structures of LGM and LGK differ. The red asterisk marks the location of the substituent change at C3 (IUPAC numbering) of the pyrazolopyridinonyl core from methyl to trifluromethyl. The yellow asterisk marks the location of the *o*-substituent change of the distal phenyl from *N*-methylpyrrolodyl to methylsulfonyl. (*b*) Placement of target ligand (green) in its electron density after graph theory was used to associate analogous atoms and adjust its conformation accordingly. The reference ligand (cyan) was carried forward from (*a*) for comparison. (*c*) Comparison of the *GLR*-placed target ligand (green) with the results from the final refinement (cyan).

Two FXa structures (Table 1 ▶) demonstrate the ability to associate atoms in the reference and target ligands, generate a reasonable conformation for the target ligand and then place the target ligand in unmodeled electron density. From the perspective of the protein, this case is straightforward (same protein; same crystal form; one protein molecule per asymmetric unit). In fact, PDB entry 3kqc was originally solved by molecular substitution (*i.e.* rigid-body refinement based on an earlier structure, with no need to run molecular replacement). Nonetheless, even in these simple cases the use of *GLR* to place the similar, but not identical, target ligand proved to be helpful. The reference ligand (LGM in PDB entry 3kqe; Fig. 6 ▶) and the target ligand (LGK in PDB entry 3kqc) have the same pyrazolopyridinonyl core as well as the same phenyltriazole at N1 (IUPAC numbering) of the core. Despite their close similarity, there are two minor changes: (i) methyl→trifluoromethyl at C3 of the core and (ii) the *o*-substituent of the N6 phenyl changed from *N*-methylpyrrolodyl to methylsulfonyl. The biological unit of FXa is comprised of one heavy chain and one light chain connected by disulfide linkages. LGM was associated with the heavy chain, so only one copy of LGK was placed in the target structure. The electron density for 3kqc was consistent with these chemical differences between LGM and LGK (Fig. 7 ▶). After real-space refinement, LGK fitted the electron density very well. The real-space correlation, as calculated with *phenix.real_space_correlation*, was 0.85 for the *GLR*-placed LGK *versus* 0.75 for the superimposed LGM (the correlations for the atoms in the replaced pyrrolodine ranged from 0.38 to 0.58). Furthermore, the conformation of LGK remained essentially unchanged after subsequent crystallo­graphic refinement (the real-space correlation was 0.84 after the final run of *phenix.refine*). Two other related structures, 3kqd and 3kqb, were also tested. For 3kqd, its ligand, LGL, is even more similar to LGK than LGM (both contain the trifluromethyl group). For 3kqb, its ligand, LGJ, is unique because the pyridinonyl ring is opened (it also differs by the addition of an *o*-fluoro atom on the interior biphenyl ring). Even so, both 3kqd and 3kqb were successfully used as both target and reference structures. Chemical changes of this nature are very common in iterative structure-based drug design (iSBDD) as chemotypes are enumerated [*e.g.* the substituent progresses from methyl, ethyl to propyl; nitrogen(s) are added to, or walked around, ring systems as in phenyl, pyridine, pyrimidine and pyrazine; ring systems are open and closed]. The need to solve many such structures of protein–ligand complexes served as the genesis of the *GLR* method (Klei *et al.*, 2011 ▶).

### 3.4. Case 2: different crystal form (one molecule in asymmetric unit of reference→two molecules in asymmetric unit of target); similar ligand   

Two p38 kinase structures (Table 2 ▶) highlight how different crystal forms and unequal numbers of protein–ligand complexes per asymmetric unit are addressed. The placement of 38P in PDB entry 3mvl based on N4D in PDB entry 3l8x proceeded successfully, much like FXa in case 1. A reference structure with one molecule in the asymmetric unit was used to complete a target structure with two molecules in the asymmetric unit; however, the roles could have been reversed. With only one instance of the reference ligand from which to choose in 3l8x, whether it contained all atoms in N4D or not, it is propagated throughout the target asymmetric unit. This propagation is fast and removes much of the tedium associated with high-copy asymmetric units. When the reference structure contains multiple copies per asymmetric unit, which copy of the ligand is selected as the reference is subject to several considerations (*e.g.* completeness, real-space correlation with electron density, average *B* factor if correlations at atomic positions are comparable). Currently, the first encountered complete, or most complete, copy of the ligand is used without regard for other considerations.

The use of *GLR* when different kinases are involved (*e.g.* the reference structure is GSK3β and the target structure is CK2) exposed one potential pitfall. With kinases, very similar ligands, and even the same ligand, can bind in different modes to different kinases because alternative hydrogen-donor and hydrogen-acceptor patterns are utilized along the hinge. In this situation, the catalytic domains would superimpose and the Tanimoto coefficient threshold would be met, but *GLR* could incorrectly place the target ligand in the target kinase. For this reason, even at the expense of some ligand similarity, it is preferable to use the same kinase for the target and reference structures. Alternatively, for structures with novel ligands and more than one active site in the asymmetric unit, the first ligand could be placed manually and *GLR* could then be used to quickly propagate it throughout the asymmetric unit.

### 3.5. Case 3: same crystal form (homodimer with single active site); mutated protein; same ligand   

Two HIV-1 protease structures (Table 3 ▶) highlight the need to check for incompatible copies of the ligand, the ability to accommodate homologous proteins and the possibility of alternate ligand conformations. Since *GLR* associates reference ligands with individual protein chains and not biological units, whenever a homomeric assembly forms an active site multiple copies of the ligand will be placed and these copies will invariably overlap. While this situation is uncommon, the HIV-1 protease homodimer, for which there are over 200 PDB entries, represents one such system. As implemented, whenever *GLR* detects incompatible ligands, only the copy with the best overall correlation to the electron density at the atomic centers is retained. In PDB entry 2fxe, DR7 (atazanavir) is bound in two alternate conformations (Klei *et al.*, 2007 ▶). Both conformations are reproduced by *GLR*; however, incom­patible copies are not currently tested as alternate conformations before being culled. When 2fxe was used as the reference structure, two possible alternate conformations existed for the reference ligand. Currently, only the first conformation is considered and propagated. For reference ligands with alternate conformations, all conformations could be sampled and propagated as dictated by the electron density at each binding site. As currently implemented, when placed ligands overlap one is discarded. Overlapped ligands could be checked to determine whether they represent alternate conformations. Handling symmetric ligands on special positions also falls under the ability to more rigorously treat overlapped ligands. As long as a meaningful structural superposition of the reference and target structures can be made based on the sequence alignment, *GLR* can be used. With mutations at only four amino acids, PDB entries 2fxd and 2fxe are easily superimposed and either can serve as the reference structure for the other. However, in 2fxd the V82F substitution results in concerted changes to the conformations of atazanavir and the protein. Consequently, in situations where many reference structures are available, the use of overall and local r.m.s.d.s may result in more judicious selection of the reference structure. Medicinal chemists often synthesize chiral inhibitors such as DR7 as racemic mixtures or even as more complicated combinations of diastereomers. Since enzymes are stereoselective, co-crystallization typically incorporates the preferred chiral arrangement. However, as implemented, the Tanimoto coefficient is insensitive to chirality. It could be augmented to cover the case in which structures with ligands of different chirality (*e.g.* enantiomers) are available in order to pick the ligand with the correct chirality as the reference.

### 3.6. Propagation of the first complete, or the most complete, version of the target ligand   

In the general case, the number of instances of the bound ligand in the reference structure, *N*_r_, and the number of instances of the bound ligand in the target structure, *N*_t_, need not be the same and can be any number greater than or equal to one. If *N*_r_ is less than one, by definition it cannot serve as a reference structure. If *N*_t_ is less than one, by definition there is no complex to refine. Usually, but not always, *N*_r_ and *N*_t_ equal the number of independent copies of the relevant protein molecule in the asymmetric unit of their respective structure. In this situation, *N*_r_ × *N*_t_ trials could be performed (*i.e.* test the target ligand morphed to match every incarnation of the reference ligand in the reference structure at every active site in the target structure). However, we felt it sufficient, and even desirable, to propagate the single best copy of the reference ligand. As currently implemented, the best copy is taken to be the first instance of the reference ligand encountered with all heavy atoms present (or the most heavy atoms present if no instance is complete). One risk with this approach is that the ligands at different active sites could be in slightly different conformations and that this site-specific behavior would be lost initially (but would hopefully be regained after crystallographic refinement informed by local electron density). However, our experience has shown that even for complicated ligands the ligand conformations across the different active sites in the asymmetric unit are often very similar and the risk of information loss is minimal. Other interesting questions were considered. For example, would it ever be advantageous to propagate a copy of the target ligand with missing atoms because it agrees better with the initial electron density? Based on many tests, the approach deemed best was to propagate the complete target ligand even if portions of certain copies needed to be subsequently deleted owing to disorder as manifested by no electron density.

### 3.7. Use in conjunction with manual ligand placement to complete an asymmetric unit   

Even if the first copy of the ligand needs to be placed manually because there is no suitable reference protein–ligand complex structure, *GLR* can still be used to propagate the ligand throughout the asymmetric unit (*i.e.* to place copies 2 through *N*, where *N* is the number of independent copies in the asymmetric unit). The crystallographer need only model the ligand in the active site associated with the most readily interpreted electron density and then use *GLR* to propagate the ligand throughout the asymmetric unit. Because of the frequency with which this use arose, the ‘replace_ligand_in_guide_pdb_file_name = true’ option was added to simplify input when the reference structure is also used as the target structure (there is no need to input an apo target structure). This option is also useful to replace problematic or incorrect ligands (*e.g.* non-standard atom names, GTP instead of ATP). The NCS_ligand functionality in *Coot* can also be used to propagate the ligand throughout the asymmetric unit. However, *GLR* can be scripted and thereby included in refinement pipelines. This approach is efficient because the difficult *de novo* placement is only performed once. Compared with the time required to place the first copy, ligand propagation throughout the asymmetric unit is fast so that the time required is much the same regardless the number of copies to be placed.

### 3.8. Integration into refinement pipelines   

**Figure 8:**
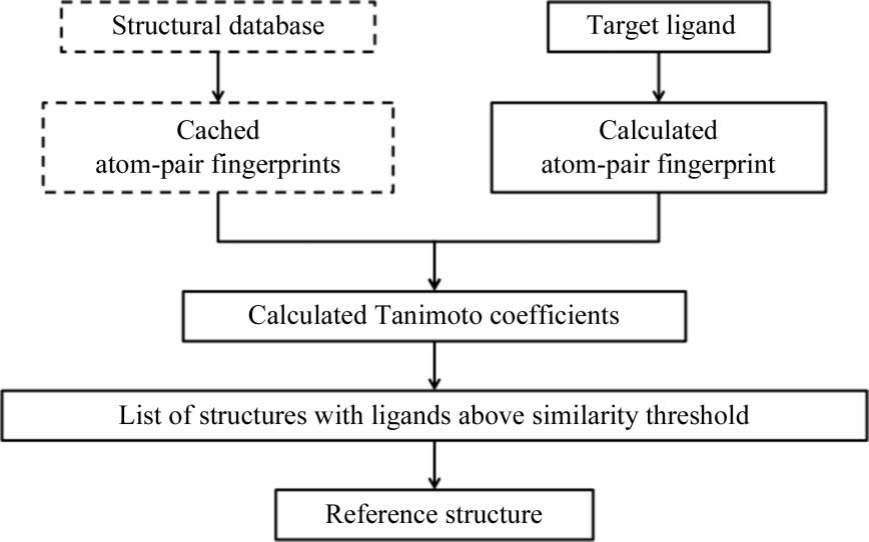
Schematic of the informatics infrastructure used to automatically provide the *GLR* reference structure, if available, as one component of the refinement pipeline. Steps outlined with dashed lines are pre-calculated. Steps outlined with solid lines are calculated for the target ligand as part of the structure refinement. A procedure based on atom pairs was used to calculate the ligand fingerprints (Carhart *et al.*, 1985 ▶). These digital fingerprints were then compared through their pairwise Tanimoto coefficient. A coefficient threshold of 0.7 was used because ligands with this degree of similarity were found to reliably bind in the expected fashion (*i.e.* with the same orientation and similar overall conformation). With diverse sets of ligands, it may be necessary to lower this threshold at the risk of false positives.

A common way to integrate *GLR* with refinement pipelines is through shell scripts and generalized PHIL instructions in which environment variables are used to define structure-specific files [*e.g.* setenv is used to define the environment variable REFERENCE-PDB for use in the PHIL instruction guide_pdb_file_name = $(REFERENCE-PDB)]. A user-written query to other databases, such as the BMSPDB (Finzel *et al.*, 2011 ▶), is also readily performed to automatically provide the reference structure (Fig. 8 ▶). The primary purpose of the BMSPDB was to provide easy access to all internal proprietary structures akin to the wwPDB with deposited structures. However, as the BMSPDB became fully implemented, another benefit emerged. In conjunction with tools like *GLR*, this curated structural database proved to be a valuable resource to assist with subsequent refinements. It is also possible to formalize the API and communicate through a defined Python object. *GLR* is especially useful when coupled with an informatics frontend to automatically provide the reference structure.

## 4. Conclusion   

*Guided Ligand Replacement* (*GLR*) enables the use of prior structures to assist ligand placement in subsequent structures. Aside from what is needed for the refinement of any protein–ligand complex, *GLR* only requires the existence of one reference structure (*i.e.* a similar protein in complex with a similar ligand). When compared with manual ligand placement and many automated approaches, the *GLR* method is fast and run time is largely independent of ligand complexity. It allows ligands to be fitted in density of poorer quality or worse resolution than is needed for *de novo* placement. *GLR* is especially useful for iSBDD work, where the structures of very similar compounds are complexed with the same target. This benefit is most apparent with complicated, flexible and macrocyclic ligands. It allows high-copy asymmetric units to be fully populated, even if the first copy of the ligand must be placed manually. It is also easily integrated into informatics-aware refinement pipelines. Much of the complexity built into *GLR* is not needed in common situations (*e.g.* a monomeric protein with a single active site and one ligand); however, this robustness was developed to make the method usable with diverse combinations of reference and target structures.

## Figures and Tables

**Table 1:** FXa: test case 1

PDB code	3kqe †	3kqc †
Role	Reference or guide	Target
Ligand	LGM	LGK
Space group	*P*2_1_2_1_2_1_	*P*2_1_2_1_2_1_
Unit-cell parameters (Å, °)	*a* = 56.7, *b* = 72.2, *c* = 77.8, α = β = γ = 90	*a* = 56.5, *b* = 72.1, *c* = 77.5, α = β = γ = 90
Molecules in asymmetric unit	1	1
Resolution (Å)	2.35	2.20

† Quan *et al.* (2010 ▶).

**Table 2:** p38 kinase: test case 2

PDB code	3l8x †	3mvl ‡
Role	Reference or guide	Target
Ligand	N4D	38P
Space group	*P*2_1_2_1_2_1_	*P*2_1_
Unit-cell parameters (Å, °)	*a* = 67.4, *b* = 71.1, *c* = 73.1, α = β = γ = 90	*a* = 67.5, *b* = 71.7, *c* = 72.8, α = 90, β = 90, γ = 90
Molecules in asymmetric unit	1	2
Resolution (Å)	2.40	2.80

† Das *et al.* (2008 ▶).

‡ Liu *et al.* (2010 ▶).

**Table 3:** HIV-1 protease: test case 3

PDB code	2fxd †	2fxe †
Role	Reference or guide	Target
Ligand	DR7 (atazanavir)	DR7 (atazanavir)
Space group	*P*2_1_2_1_2_1_	*P*2_1_2_1_2_1_
Unit-cell parameters (Å, °)	*a* = 53.4, *b* = 58.2, *c* = 61.3, α = β = γ = 90	*a* = 51.2, *b* = 58.2, *c* = 61.3, α = 90, β = 90, γ = 90
Molecules in asymmetric unit	2	2
Resolution (Å)	1.60	1.80

† Klei *et al.* (2007 ▶).
